# Supplementary material for: Investigation of the Anti-Methicillin-Resistant Staphylococcus aureus Activity of (+)-Tanikolide- and (+)-Malyngolide-Based Analogues Prepared by Asymmetric Synthesis
Source: Int J Mol Sci. 2021 Jun 15;22(12):6400. doi: 10.3390/ijms22126400 (PMC8232695; doi:10.3390/ijms22126400)
Supplement: Supplementary file 1 [file ijms-22-06400-s001.zip › ijms-1158985-supplementary.pdf]

Supplementary Materials

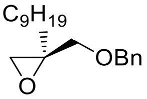

14

Reference Sample: The chromatogram of **14** was compared to a previously prepared sample of the (*S*)-enantiomer of the same compound. (Conditions: Waters Acquity UPC[2], Chiracel IB, $scCO_2$/isopropanol = 95:5, flow rate = 2 mL min^{-1}).

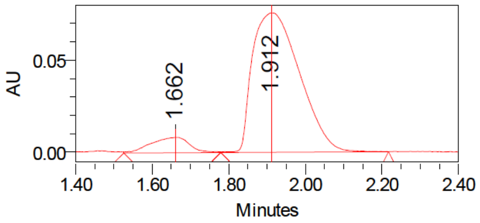

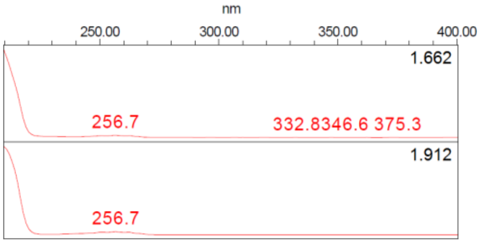

Intermediate 14: Only one peak was observed in the chromatogram of **14** shown below indicating an *ee* over 99 %. The product was assigned as the (*R*)-enantiomer due to the retention time.

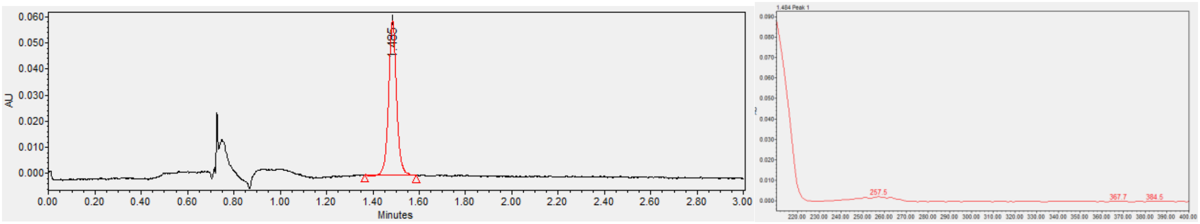

Comparison was also made to a chromatogram obtained from a previous synthesis of intermediate **14** in which a slightly lower *ee* was observed. In this case, the small peak for the (*S*)-enantiomer is visible.

Figure S1. SFC Chromatograms of Reference and Intermediate 14

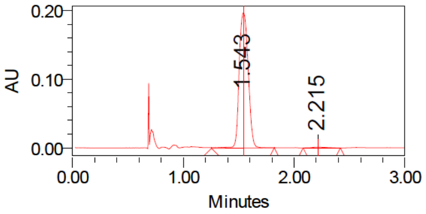

Peak Results

	RT	% Area
1	1.543	99.16
2	2.215	0.84

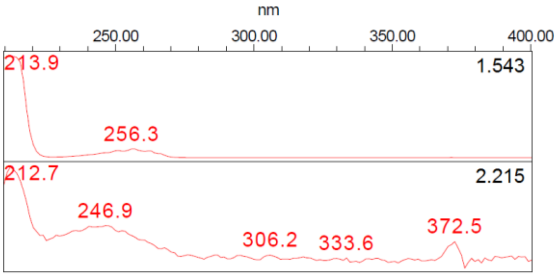

Figure S2: ^{1}H NMR and ^{13}C NMR Spectra of Compound 10

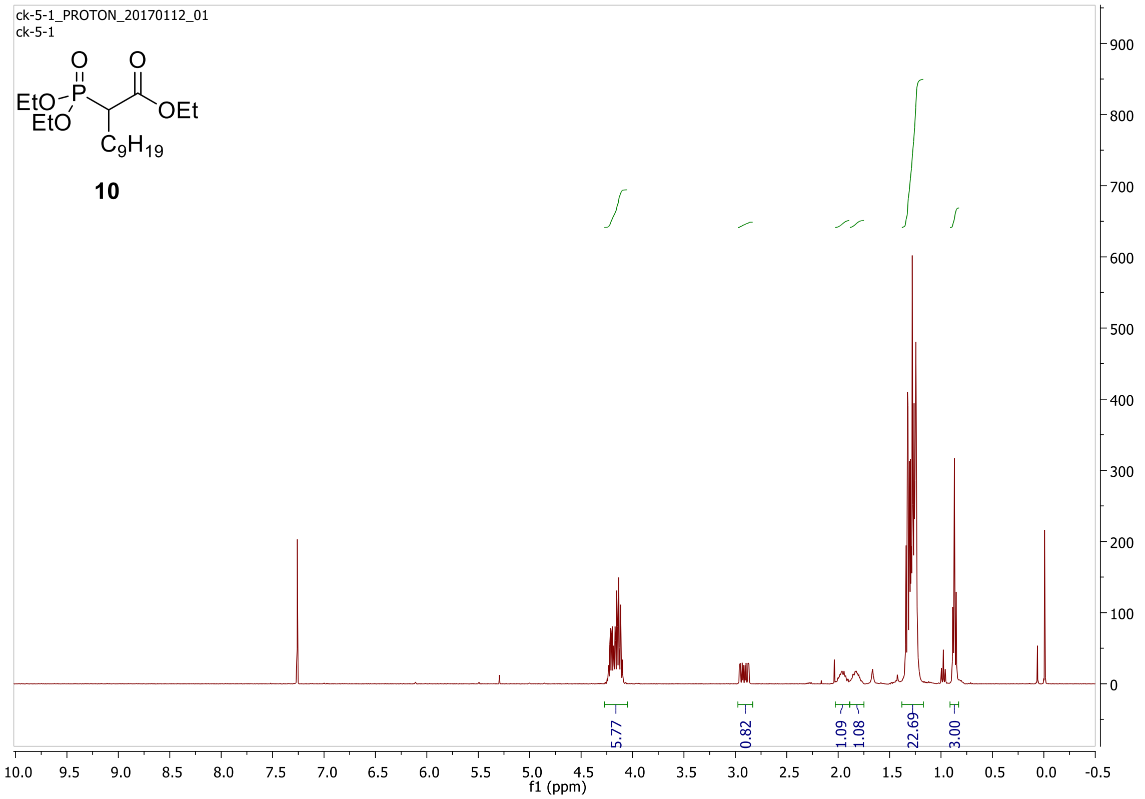

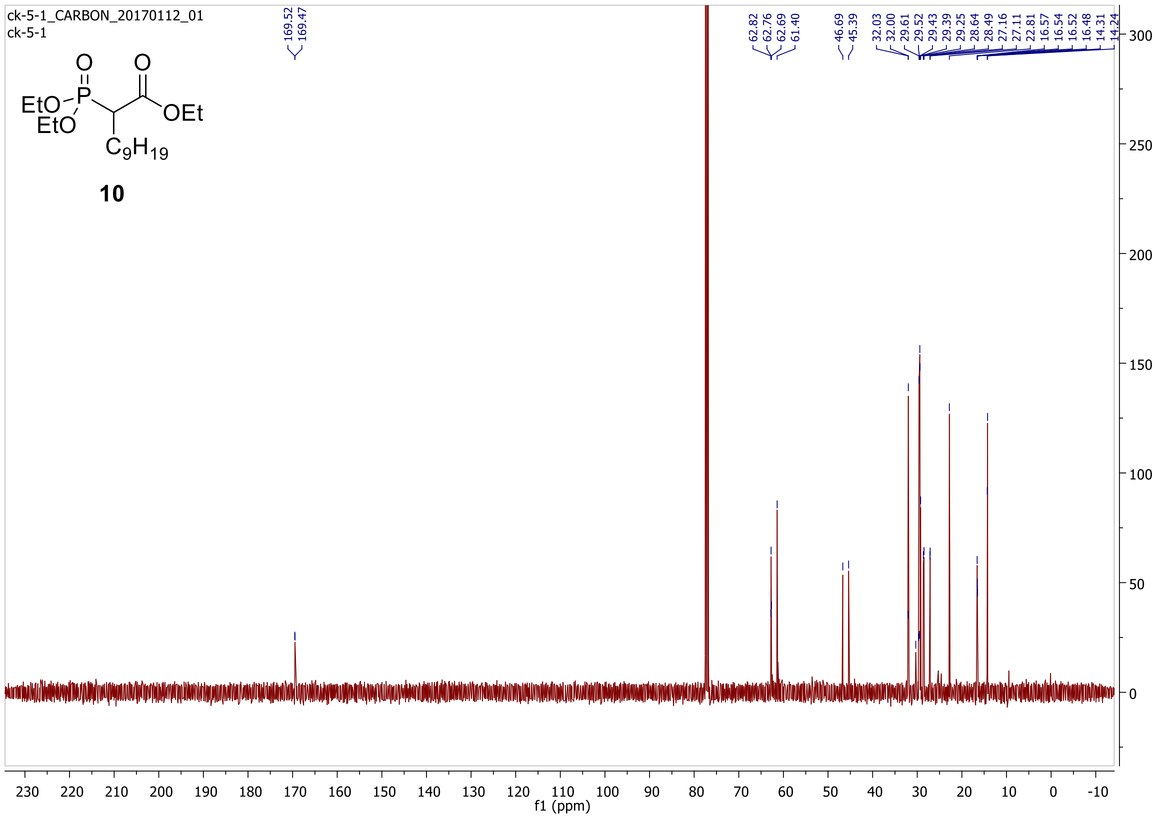

Figure S3: ^{1}H NMR and ^{13}C NMR Spectra of Compound 11

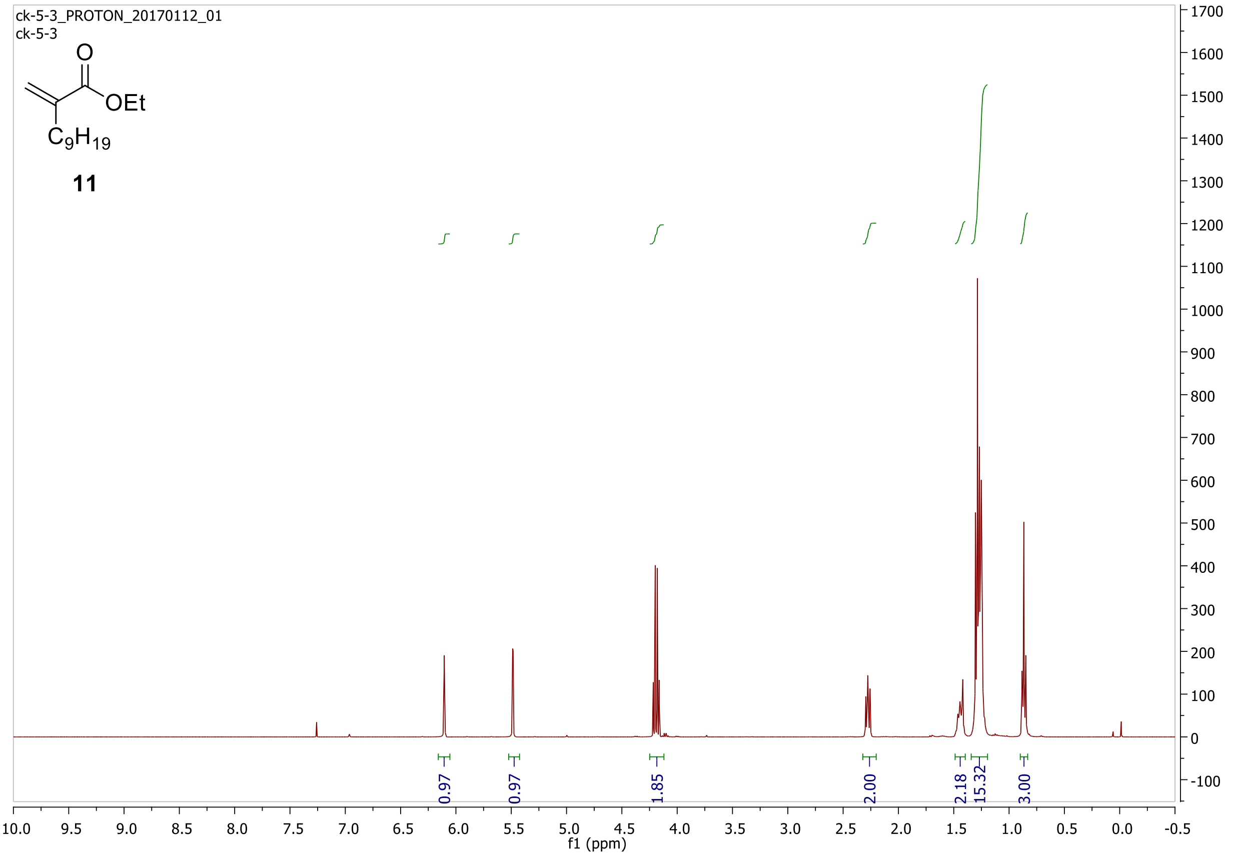

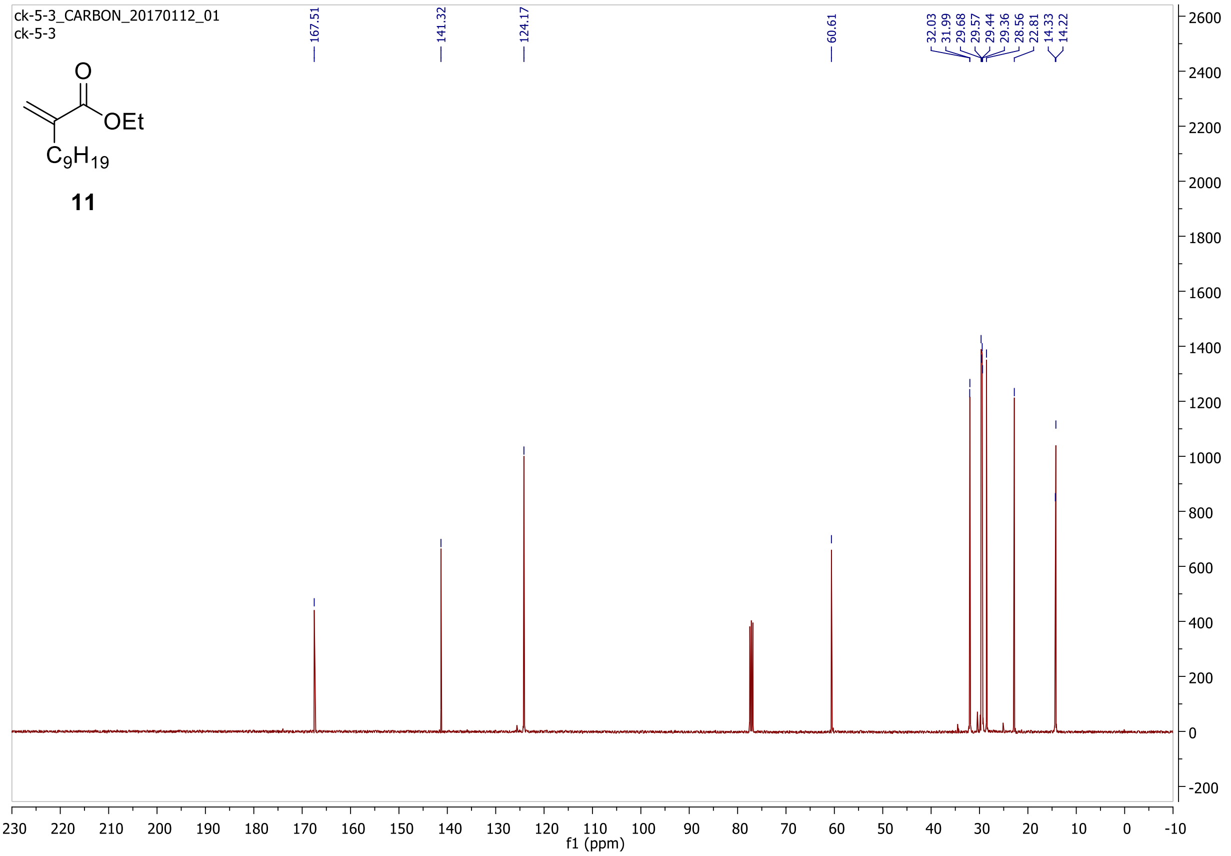

Figure S4: ^{1}H NMR and ^{13}C NMR Spectra of Compound 12

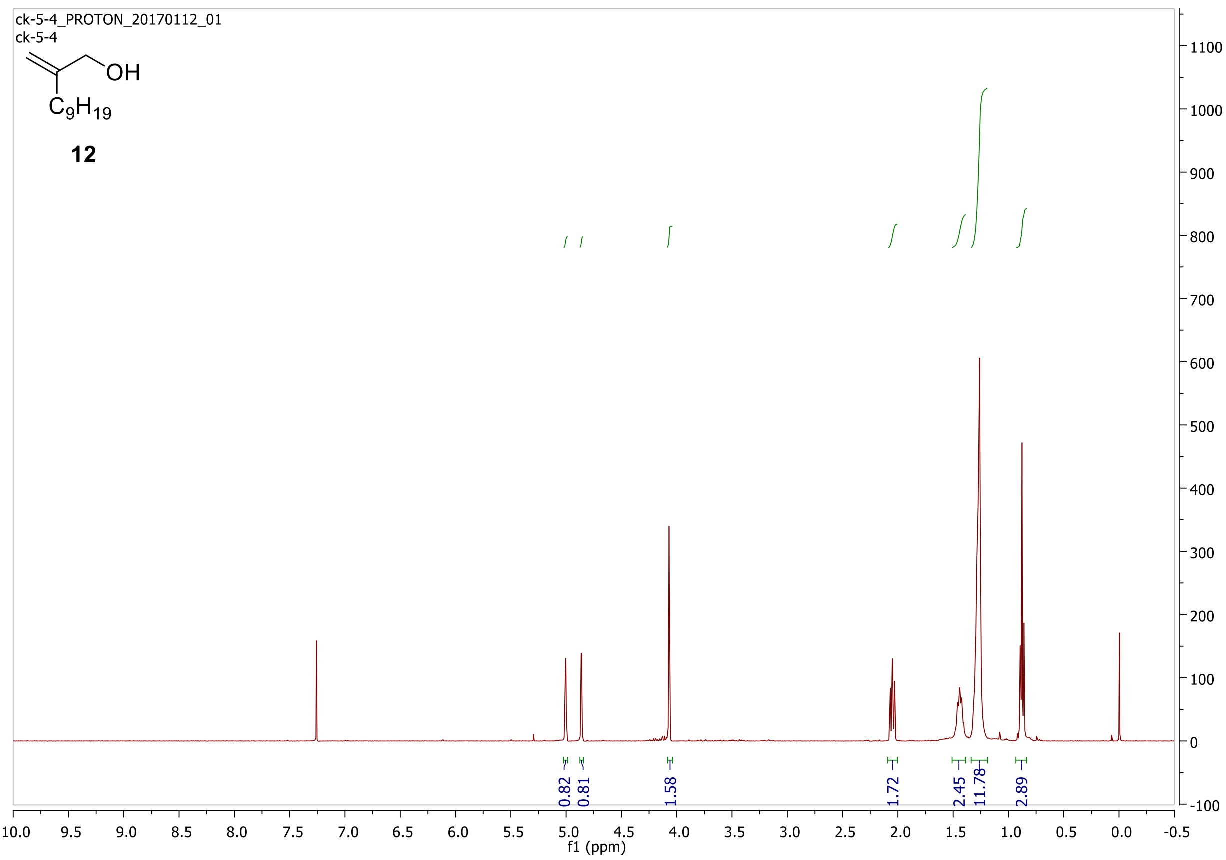

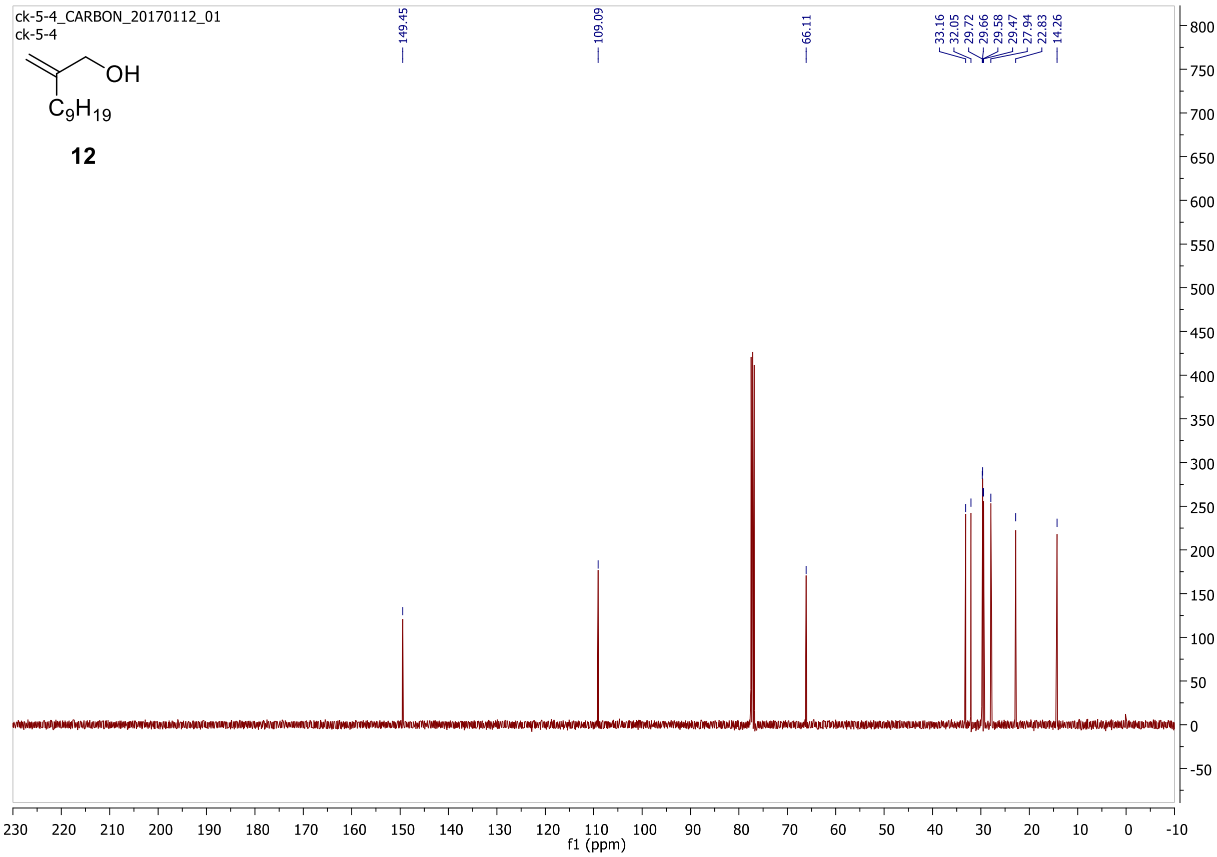

Figure S5: ^{1}H NMR and ^{13}C NMR Spectra of Compound 13

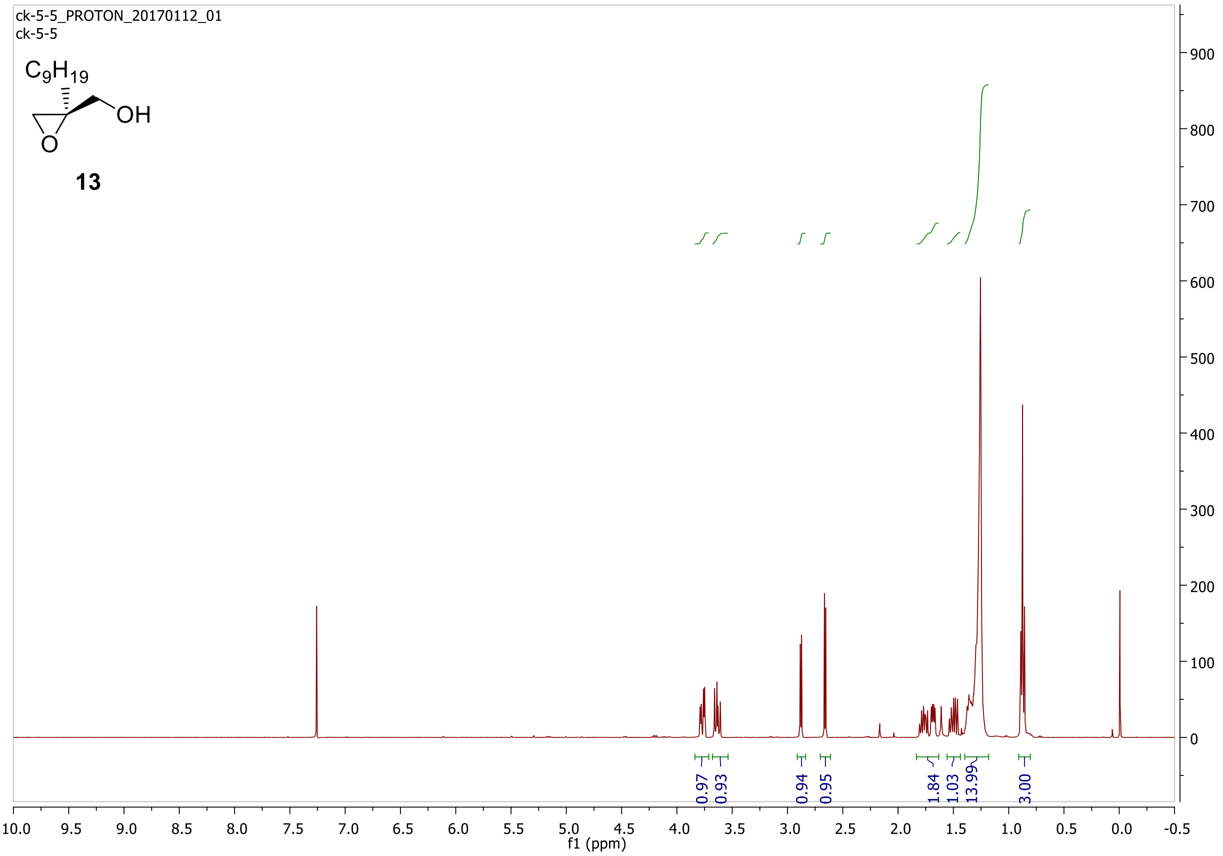

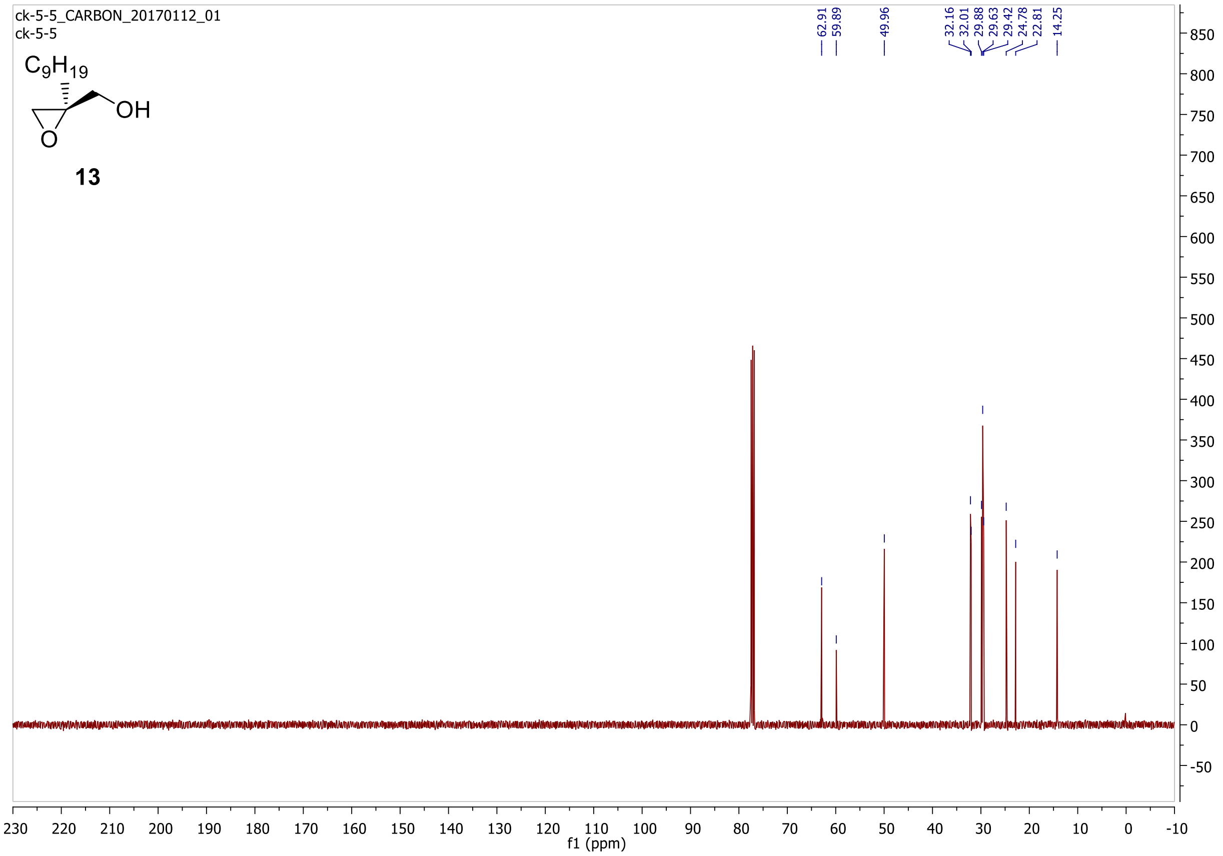

Figure S6: ^{1}H NMR and ^{13}C NMR Spectra of Compound 14

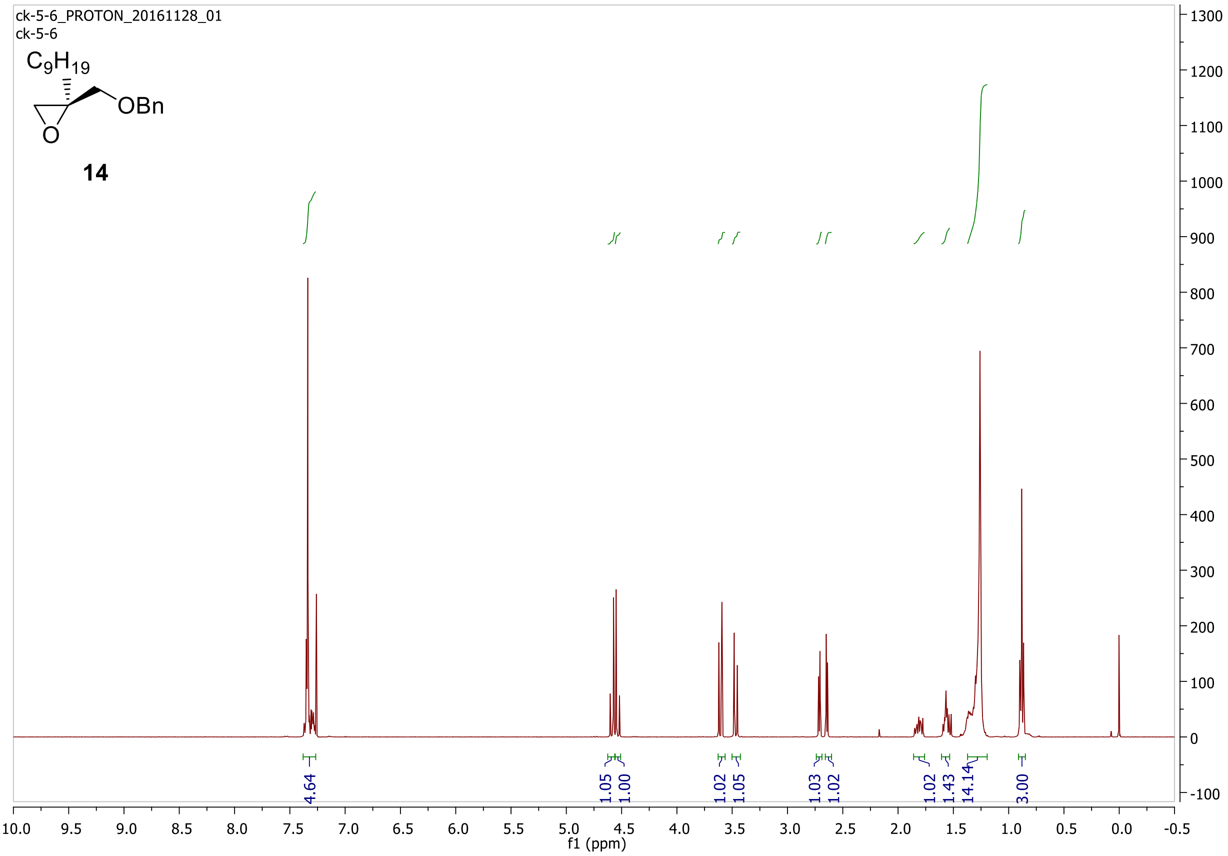

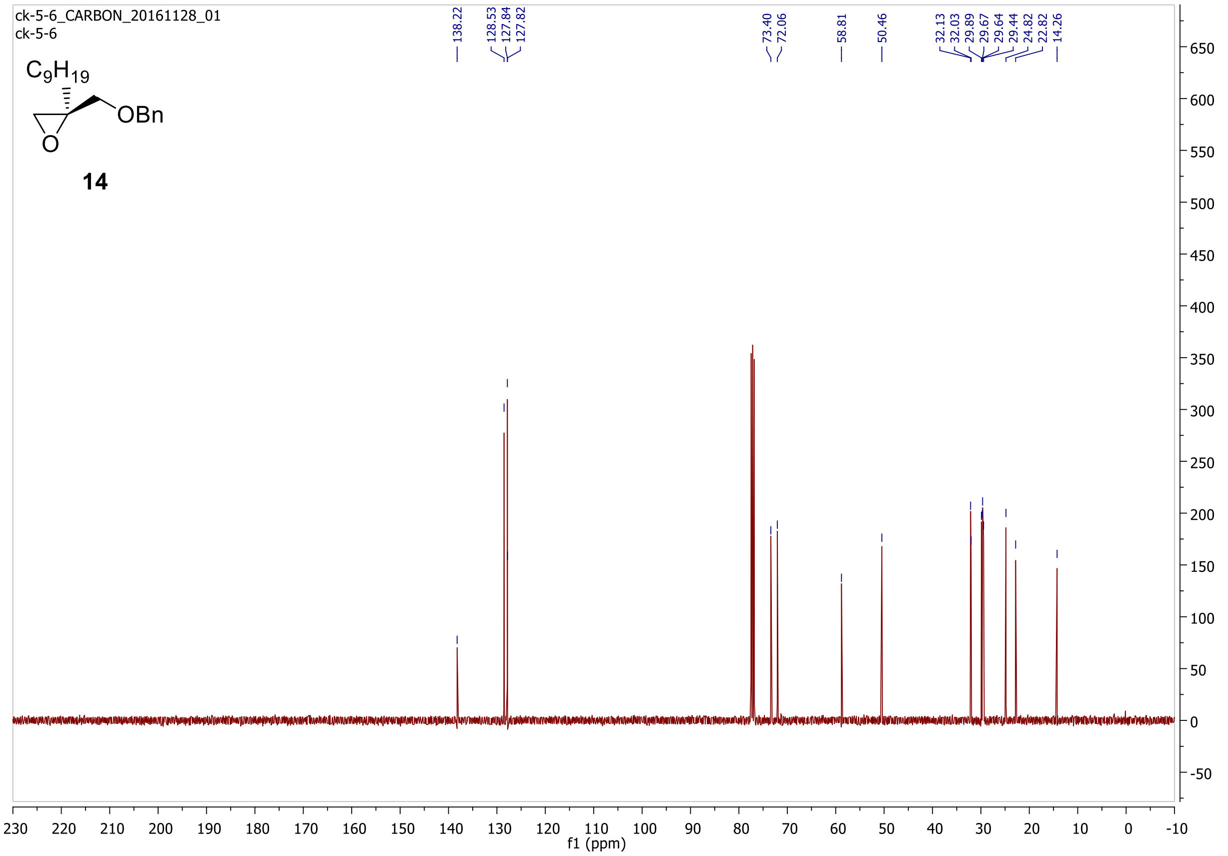

Figure S7: ^{1}H NMR and ^{13}C NMR Spectra of Compound 15

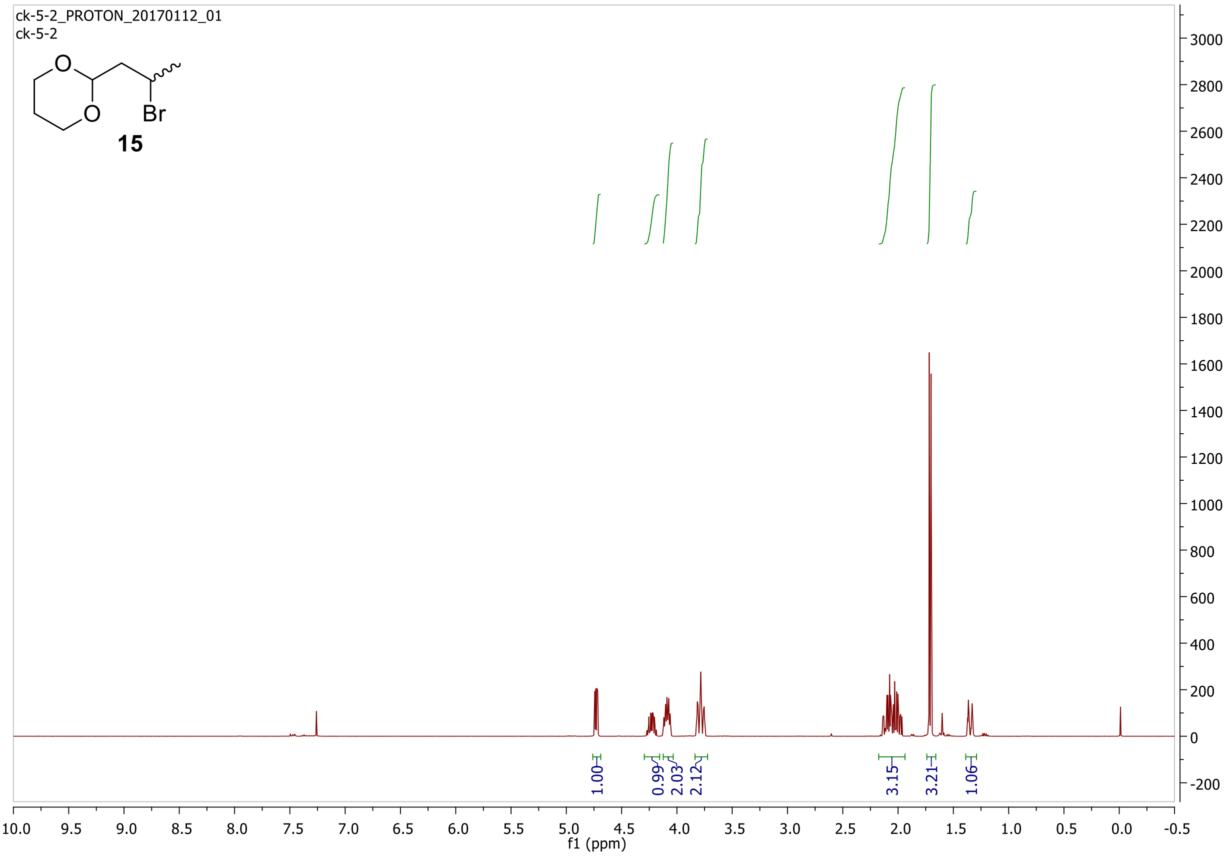

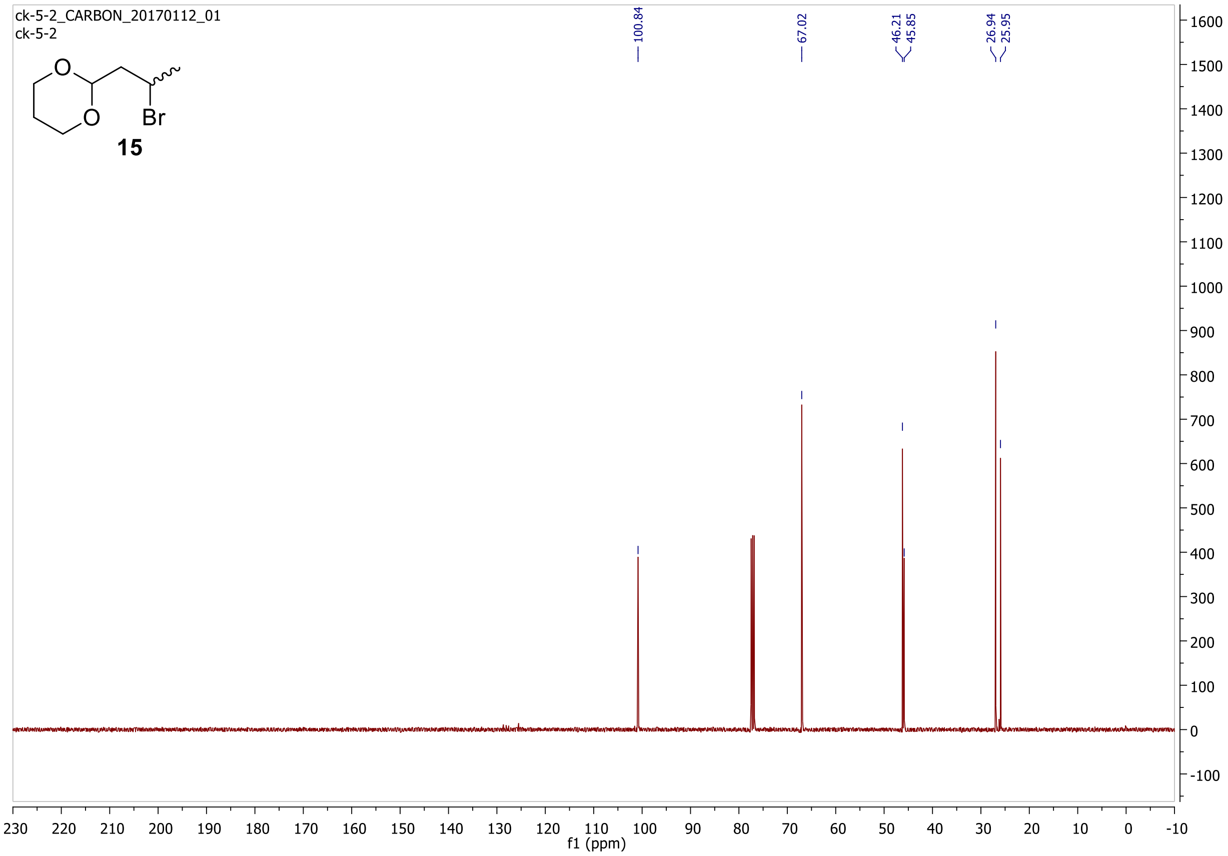

Figure S8: ^{1}H NMR and ^{13}C NMR Spectra of Compound 18

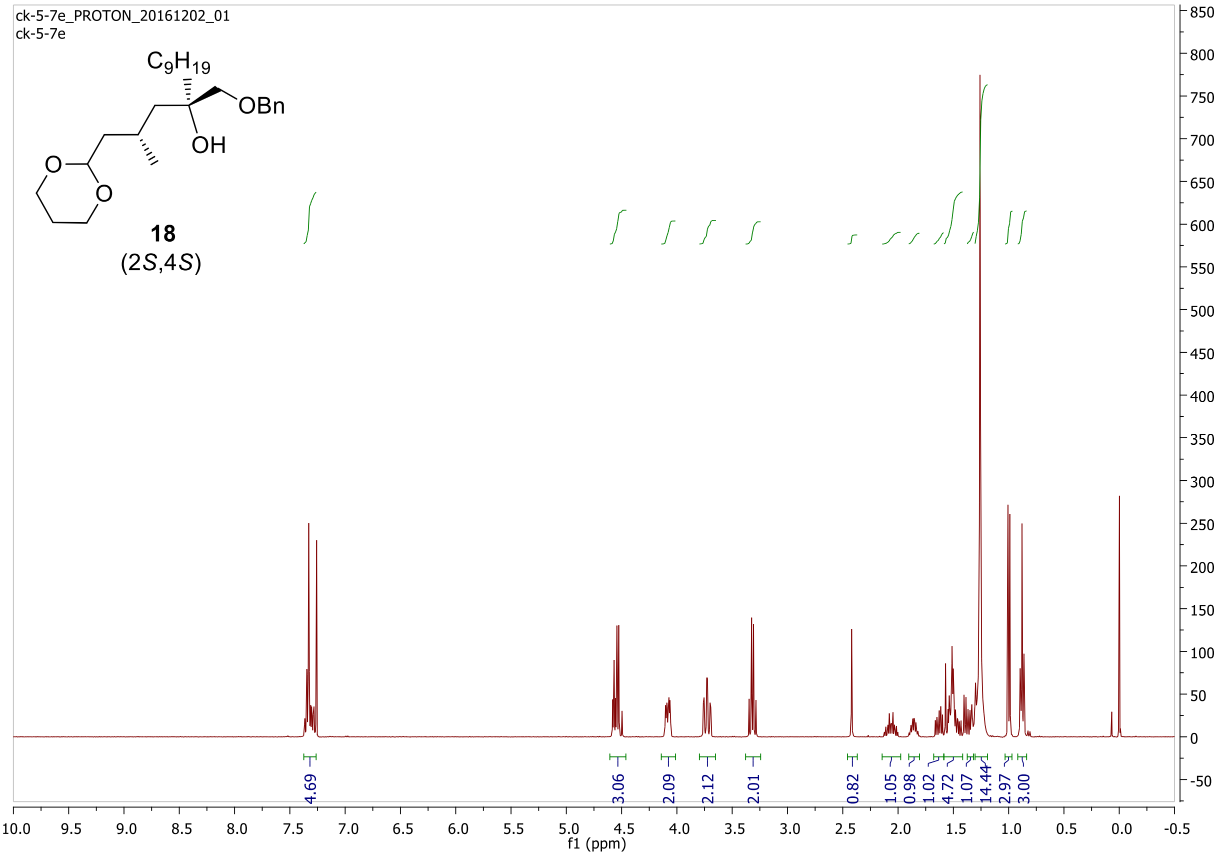

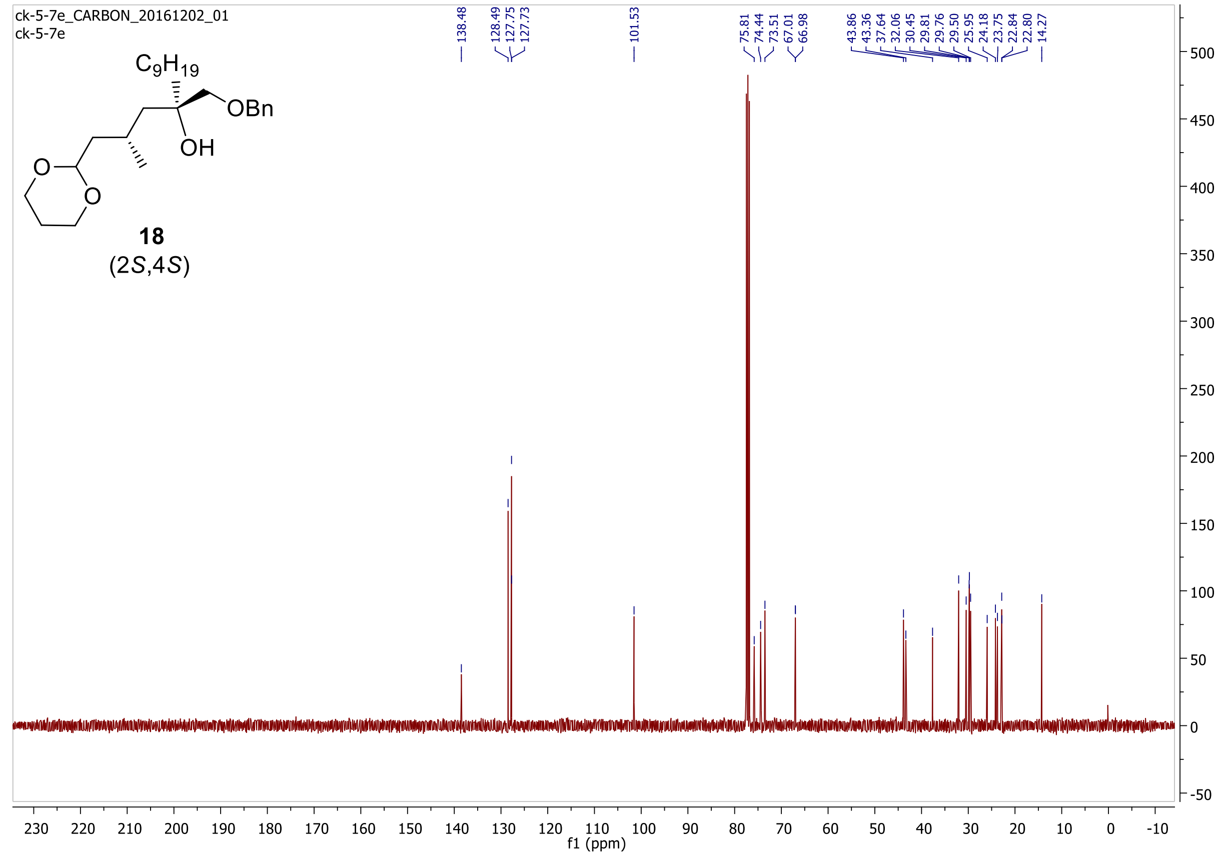

Figure S9: ^{1}H NMR and ^{13}C NMR Spectra of Compound 22

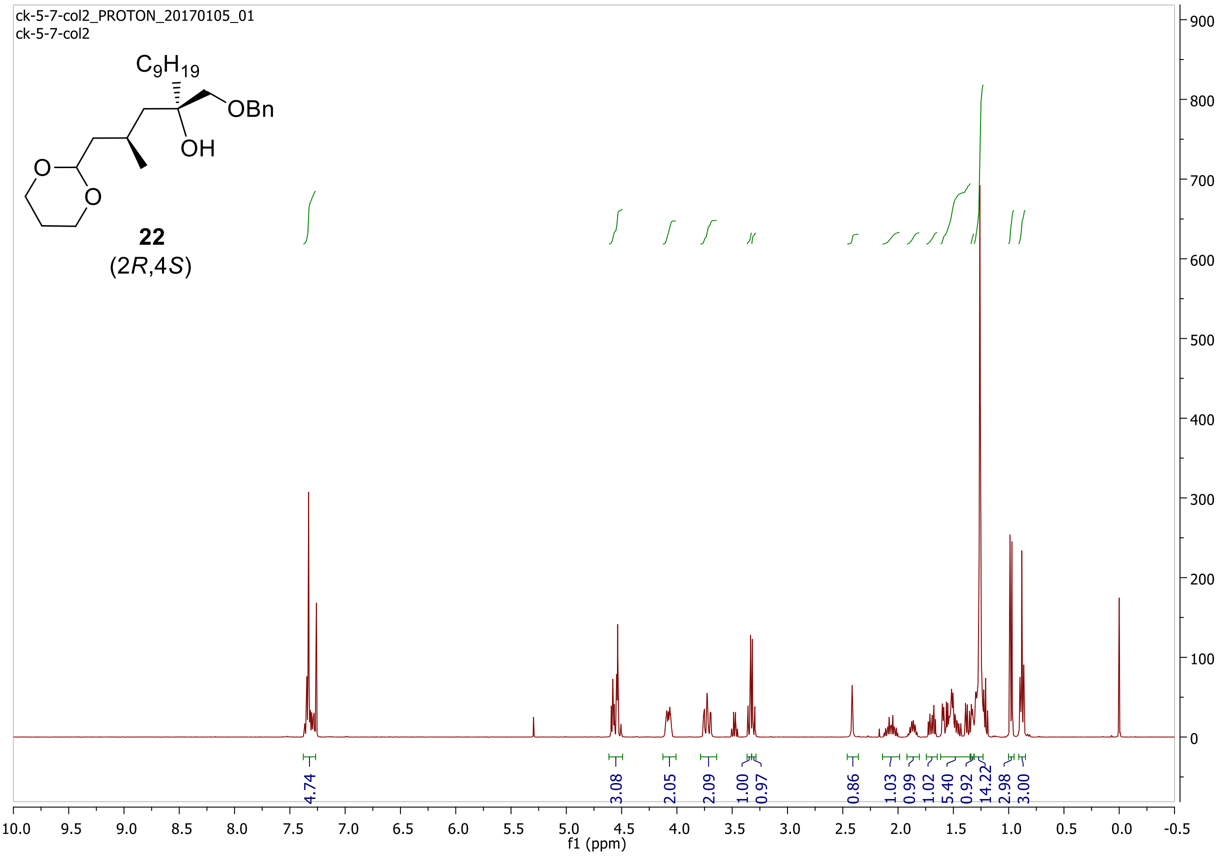

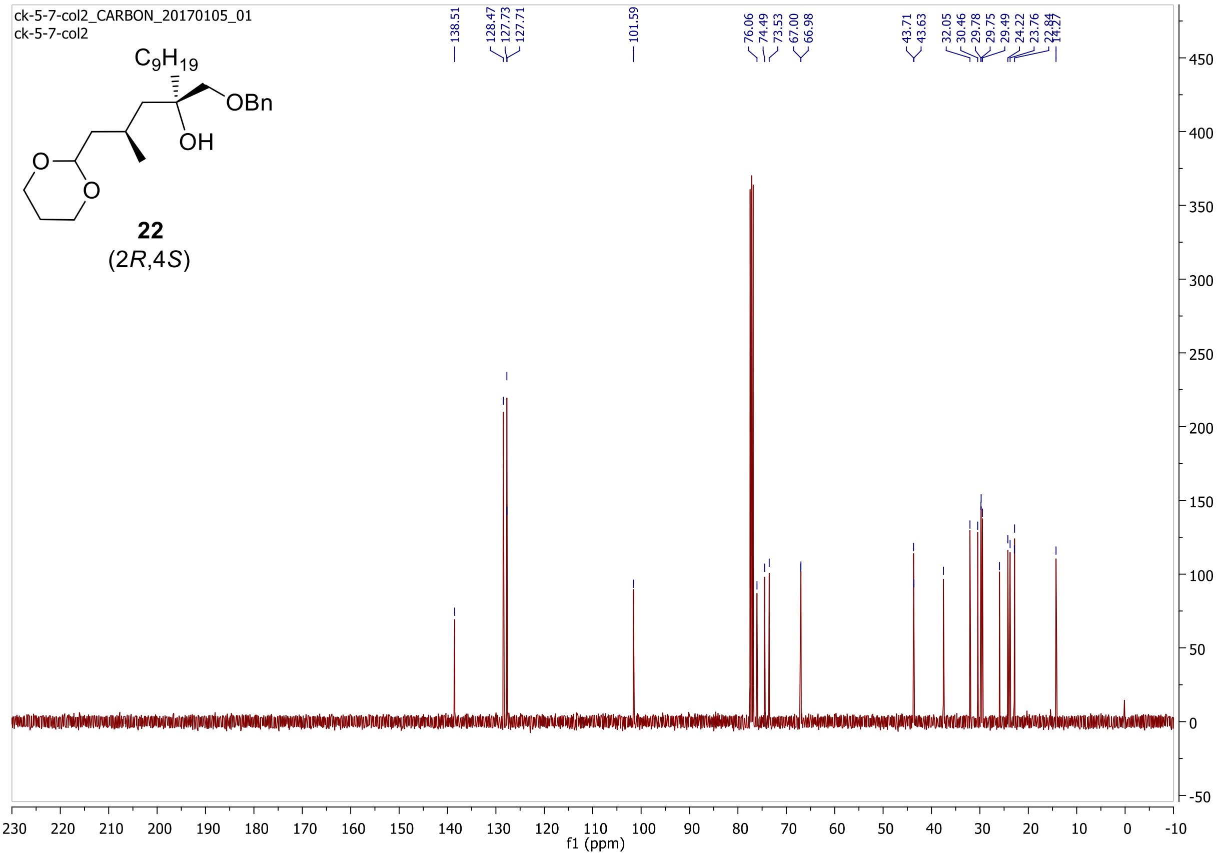

Figure S10: ^{1}H NMR and ^{13}C NMR Spectra of Compounds 23/24

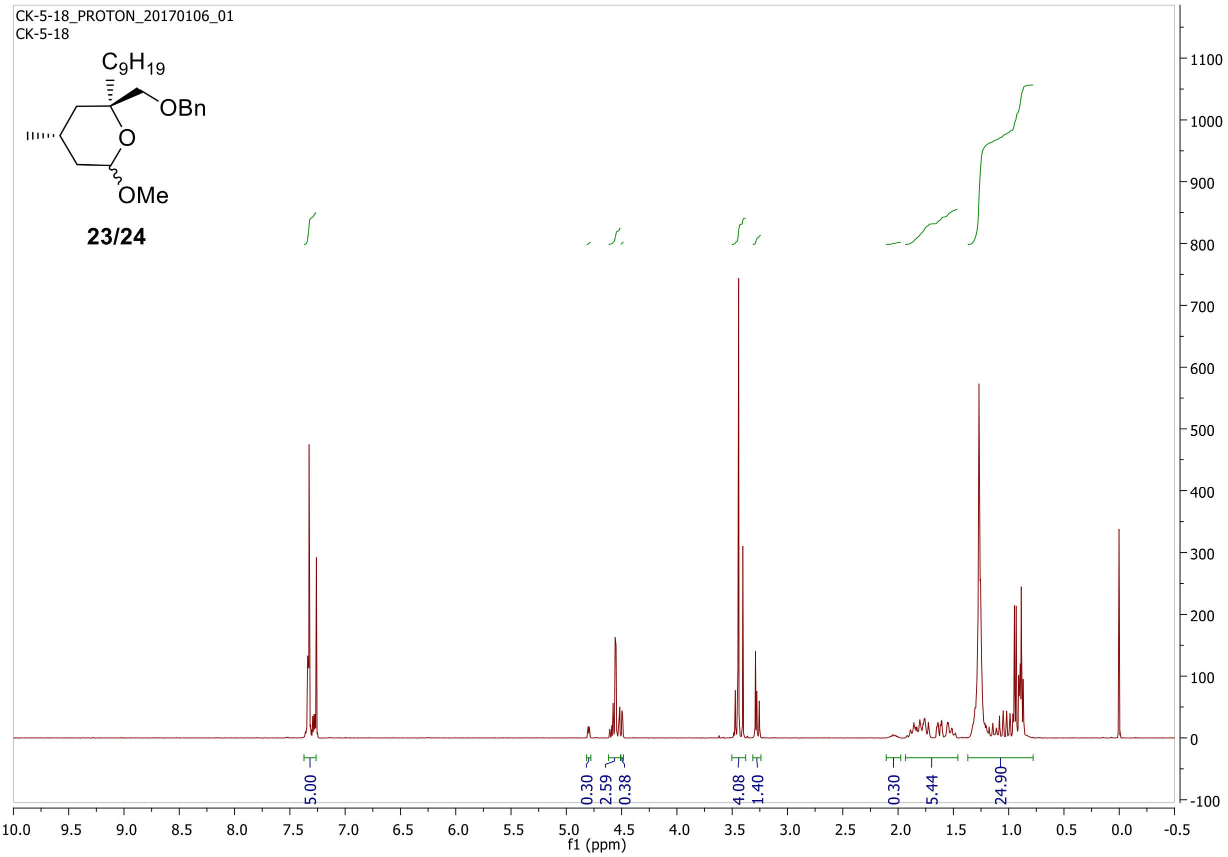

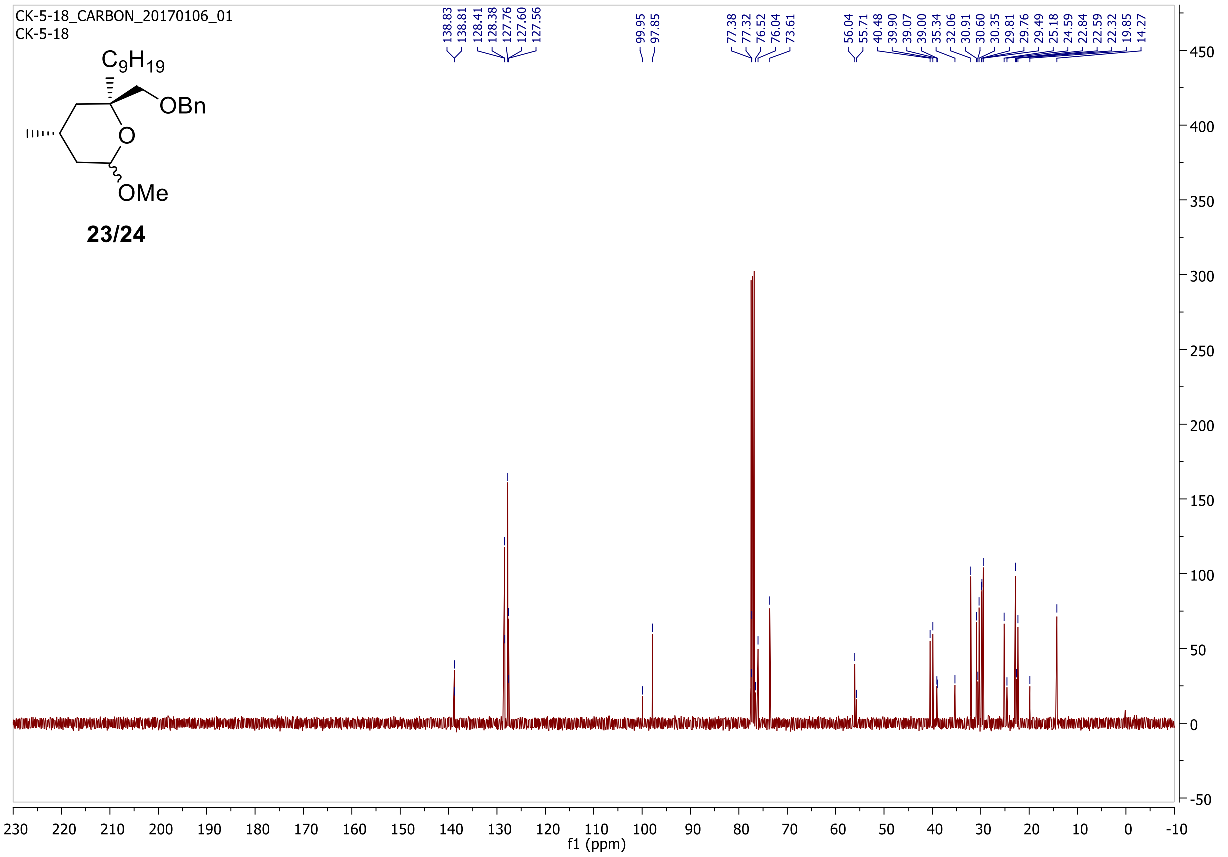

Figure S11: ^{1}H NMR and ^{13}C NMR Spectra of Compounds 19/20

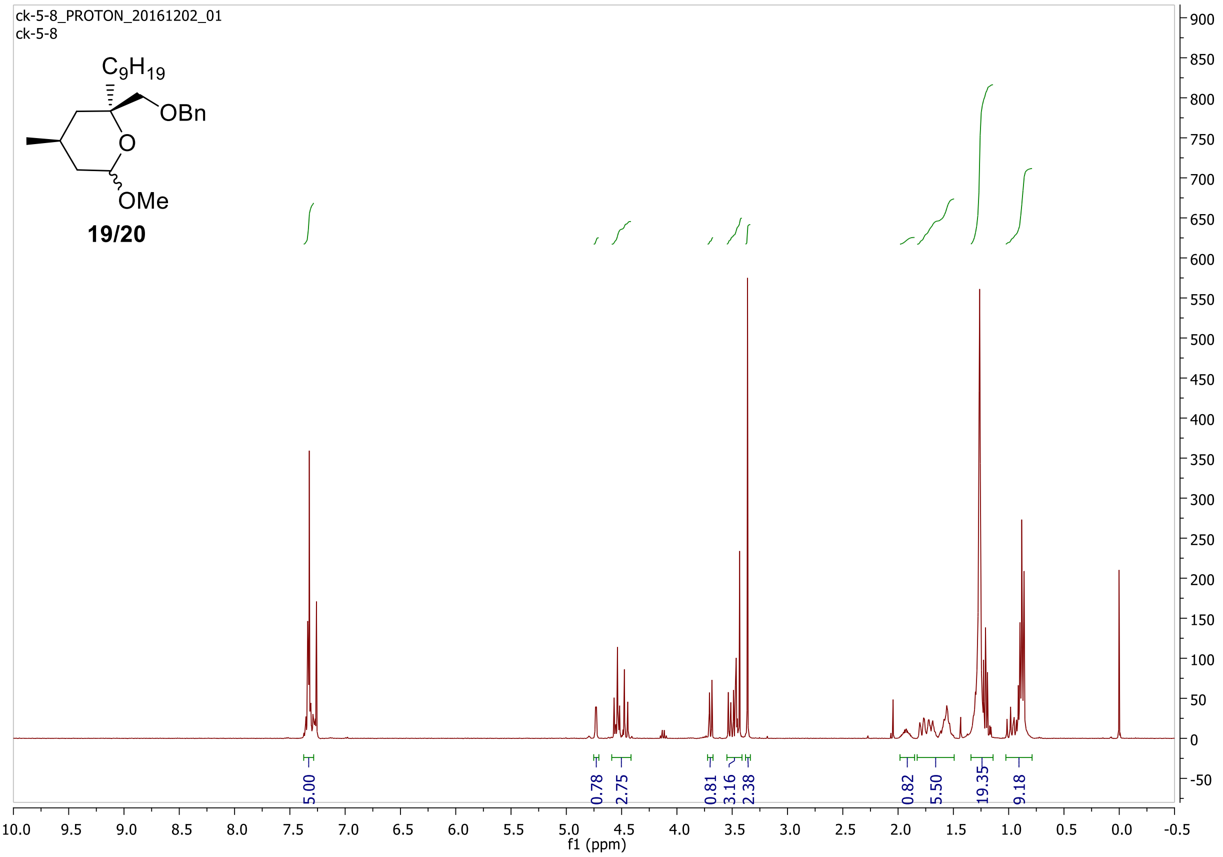

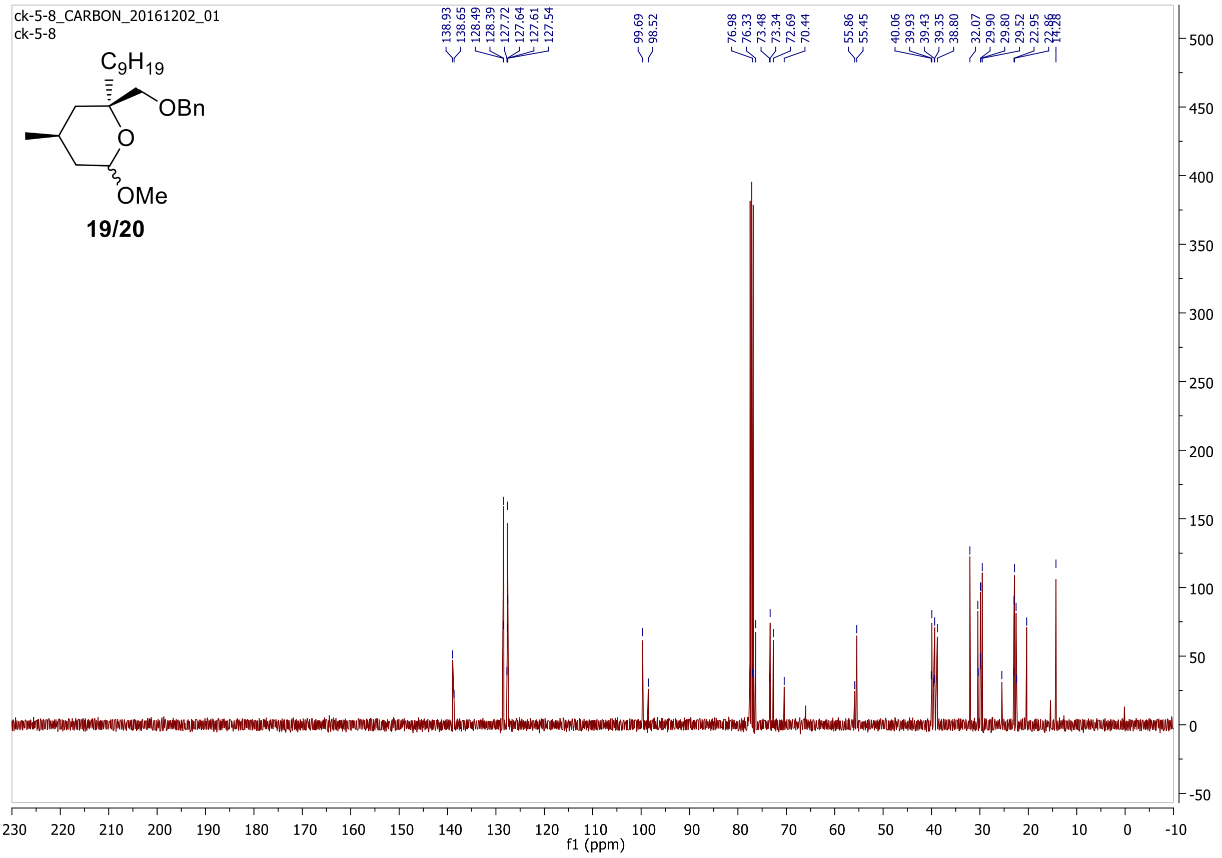

Figure S12: ^{1}H NMR and ^{13}C NMR Spectra of Compound 25

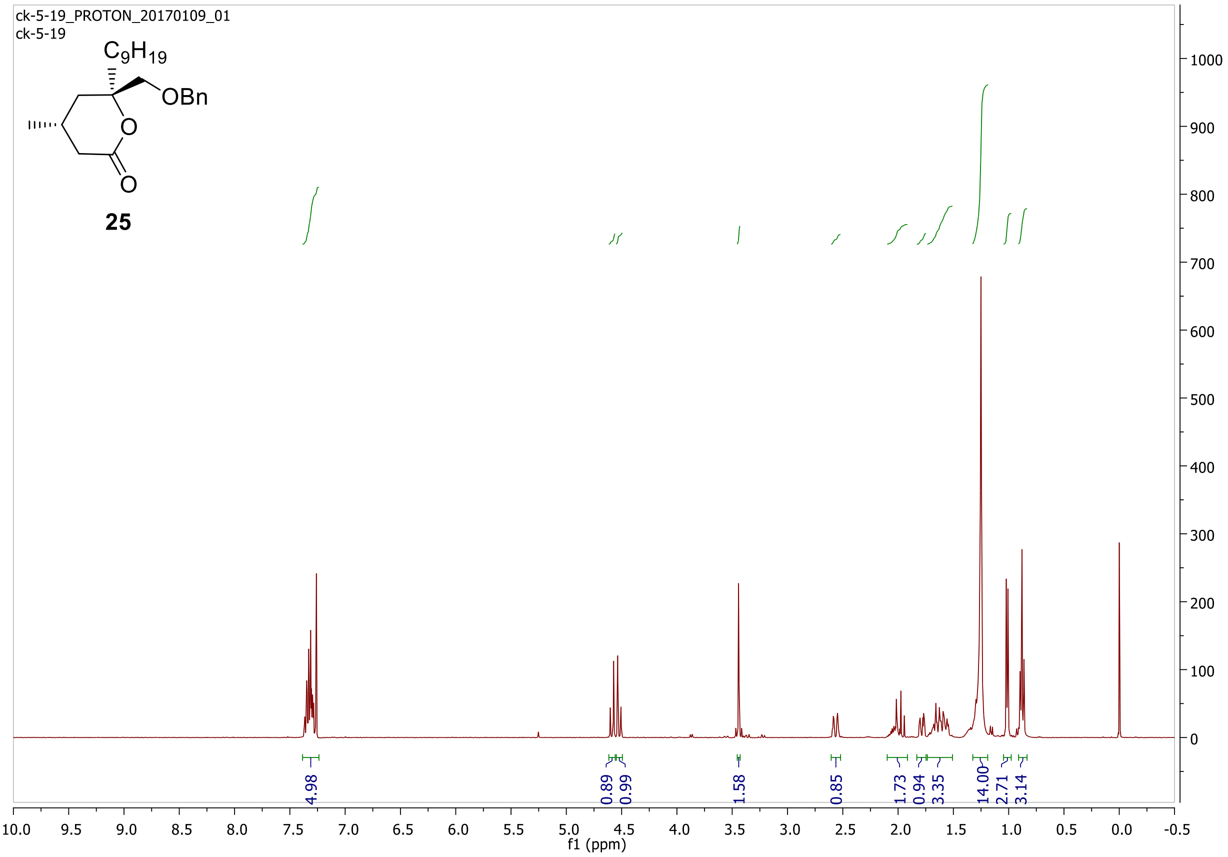

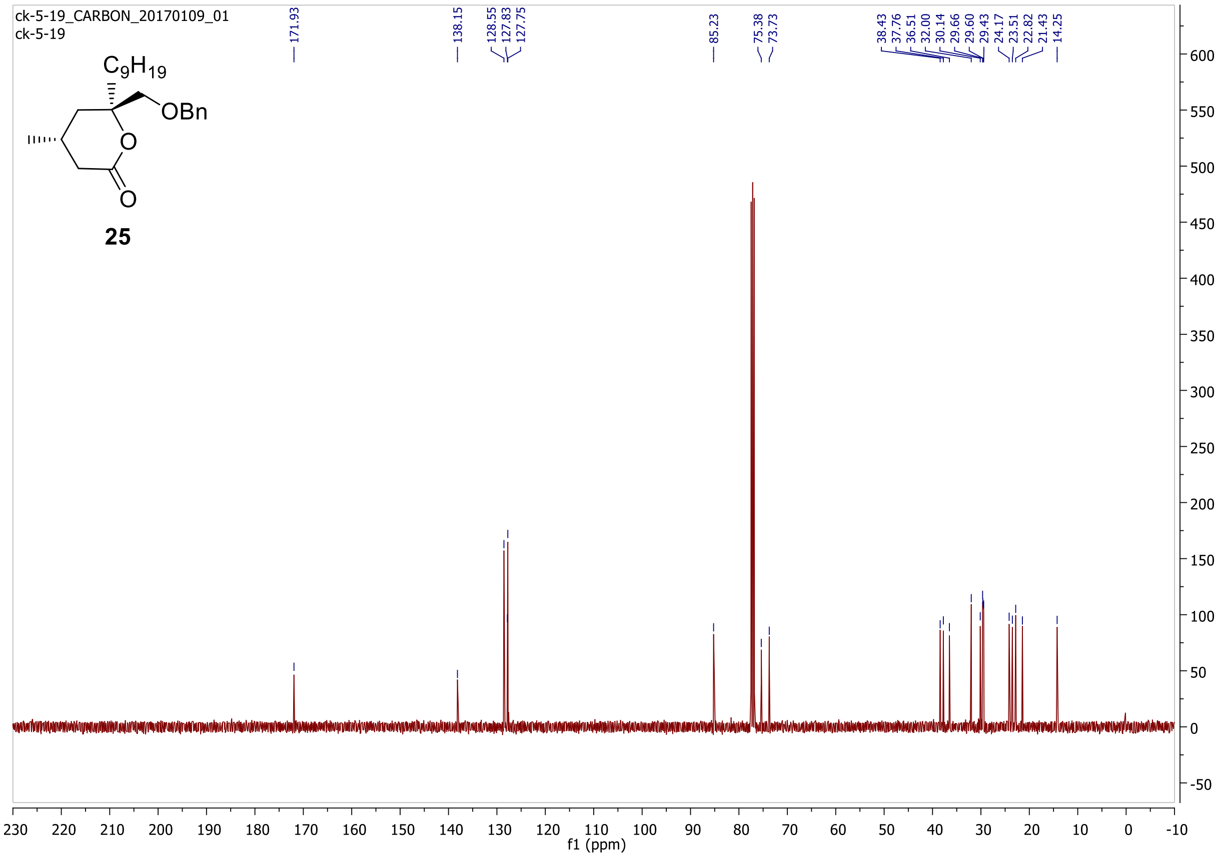

Figure S13: ^{1}H NMR and ^{13}C NMR Spectra of Compound 21

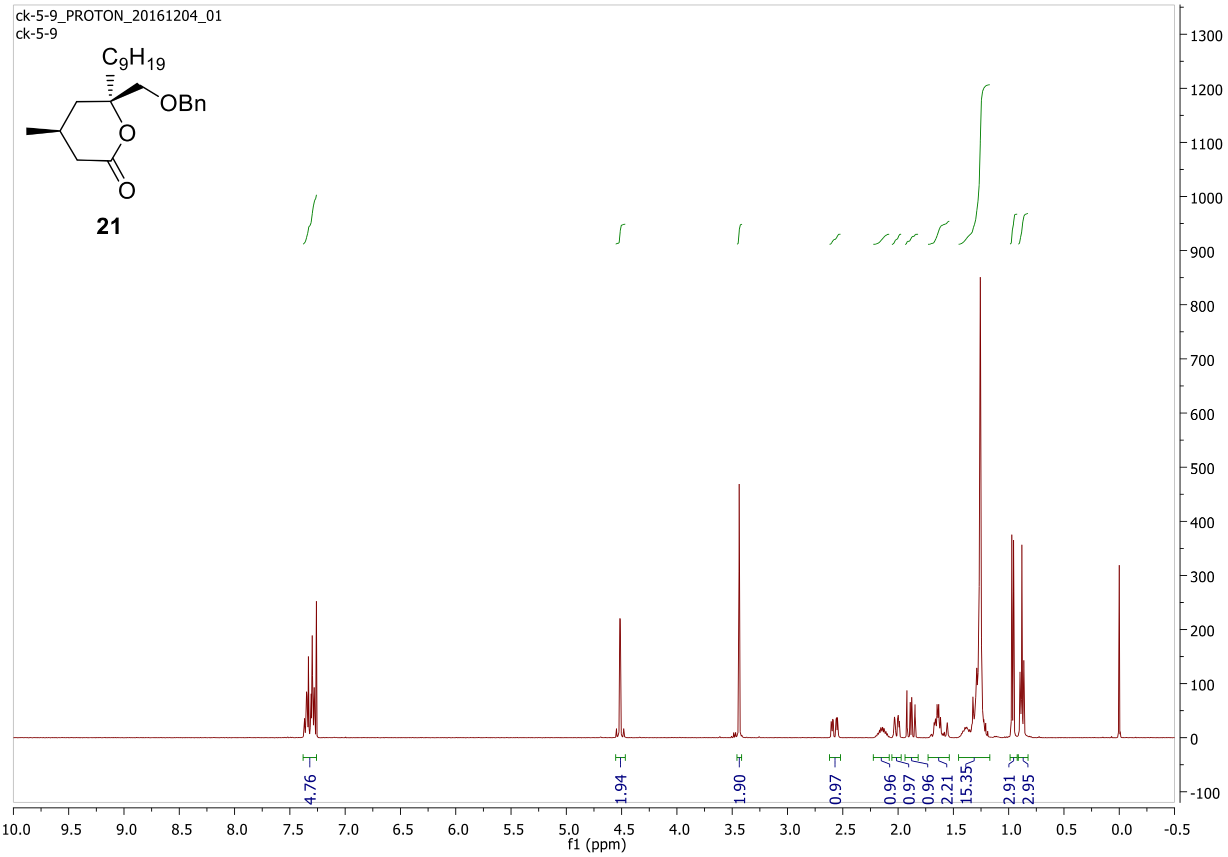

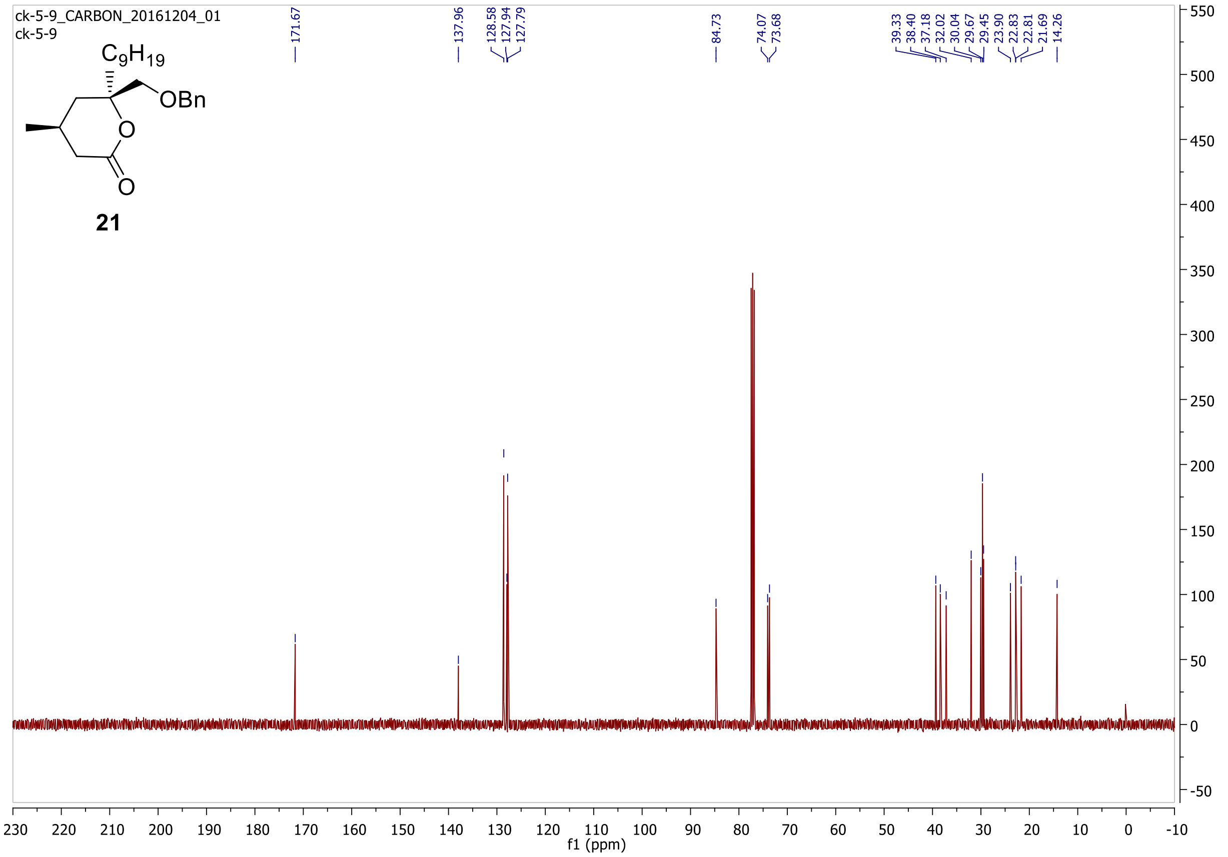

Figure S14: ^{1}H NMR and ^{13}C NMR Spectra of Compound 8

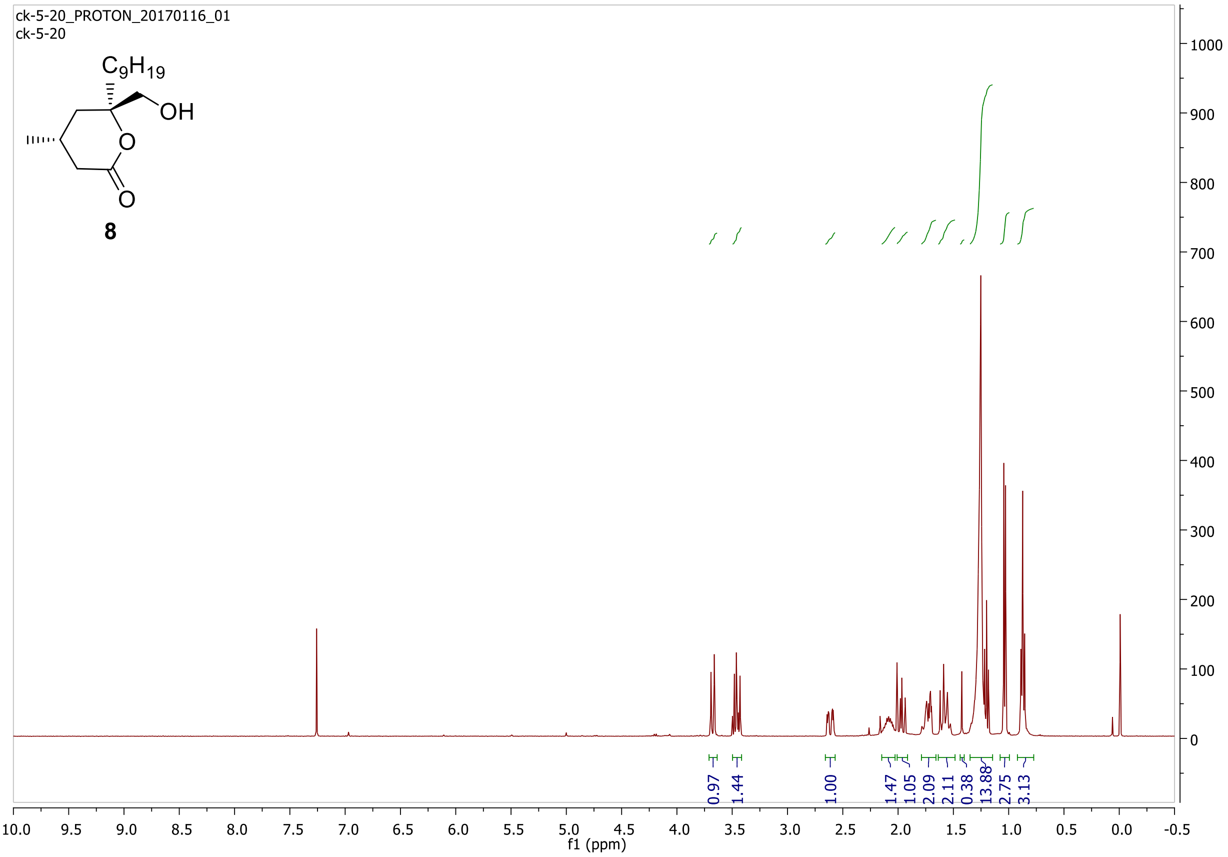

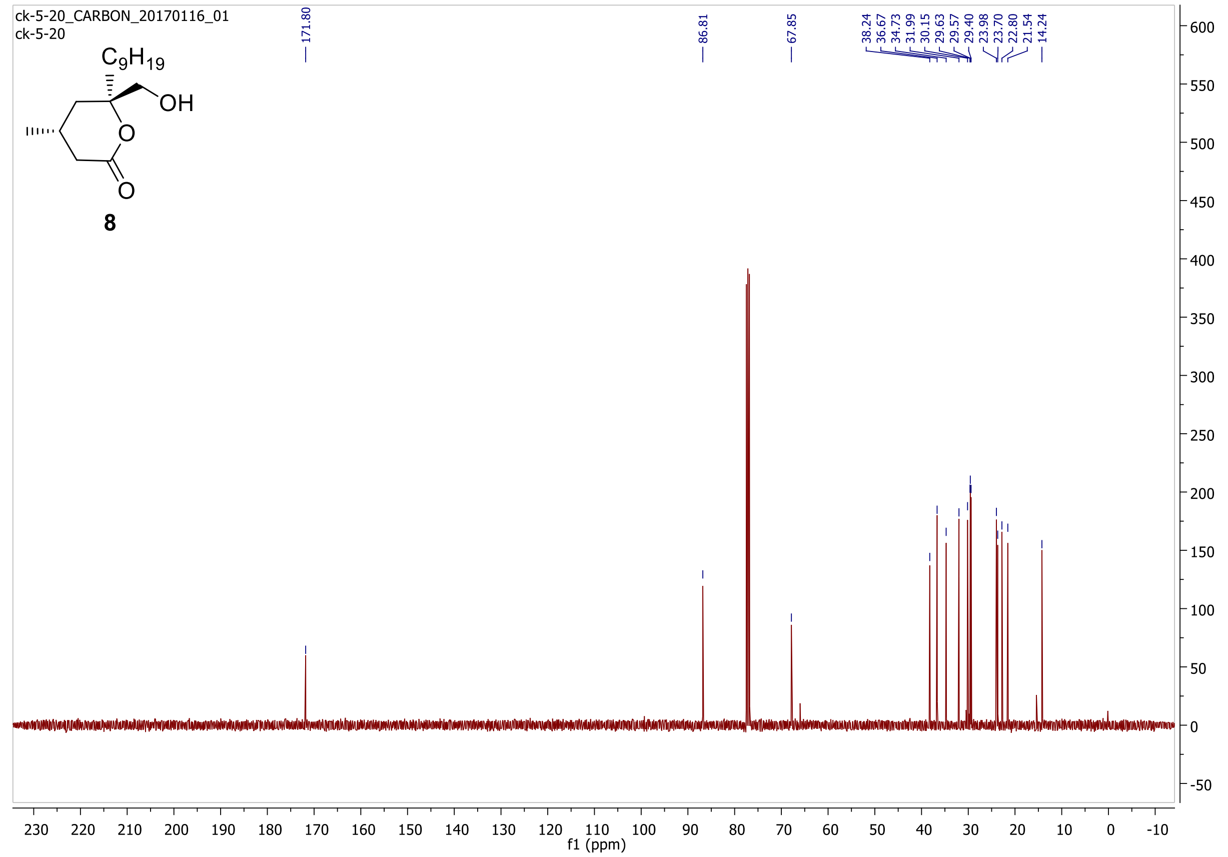

Figure S15: ^{1}H NMR and ^{13}C NMR Spectra of Compound 7

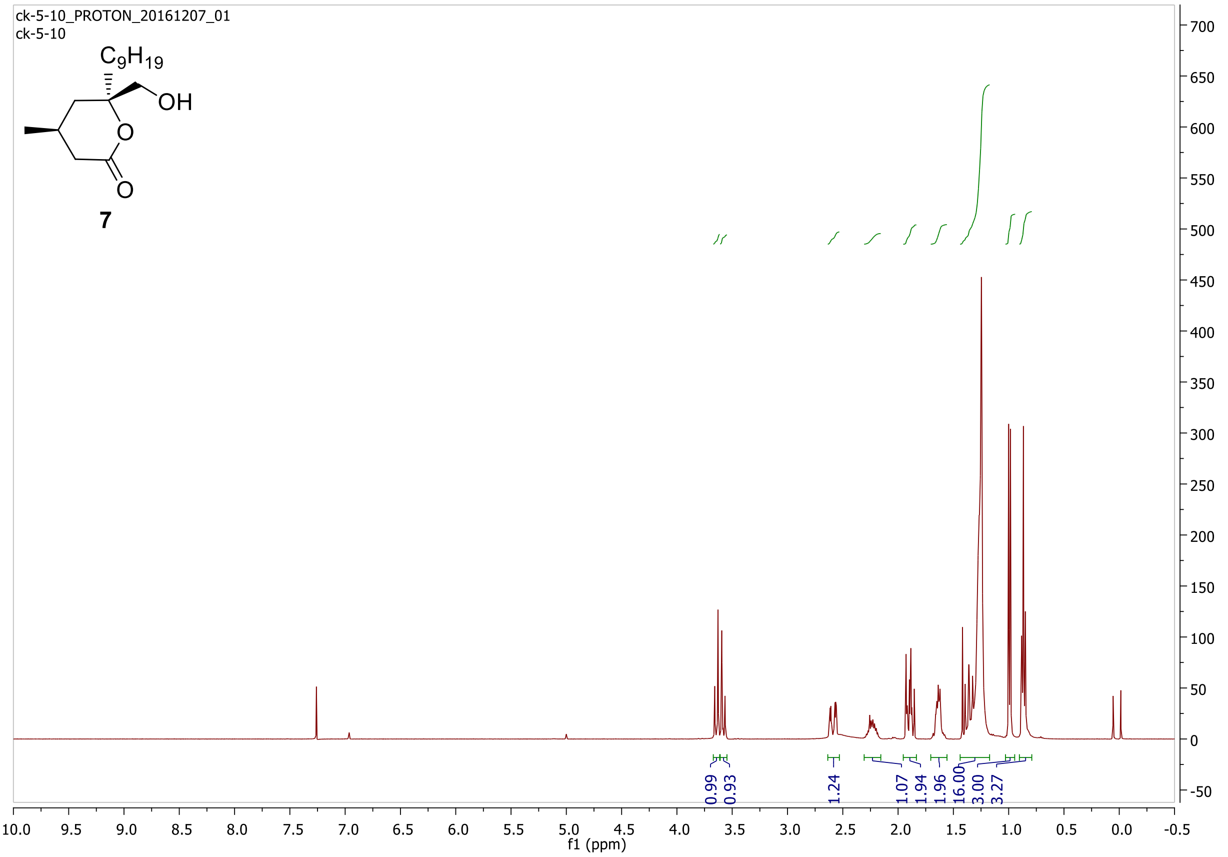

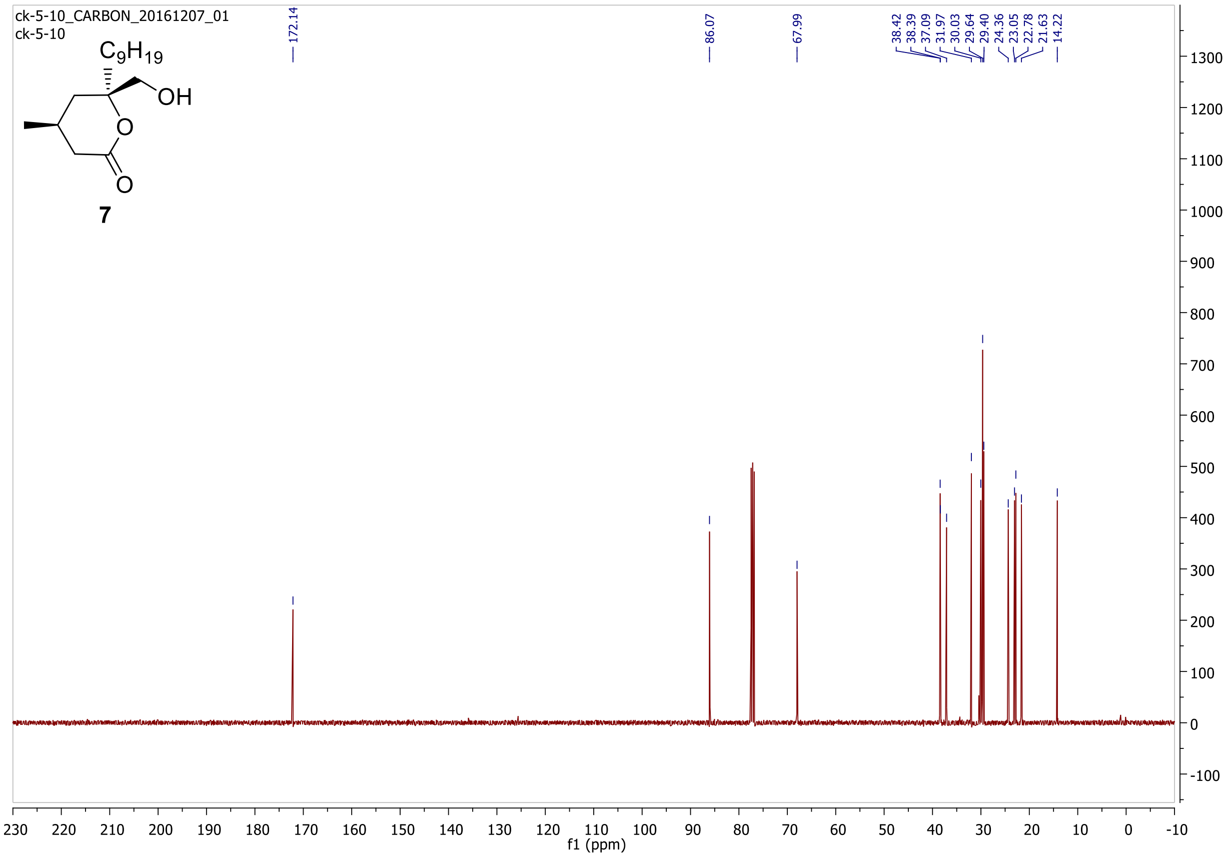

Biological Testing

Antimicrobial testing of compounds

Preparation of compounds. Samples were reconstituted into an appropriate volume of DMSO to achieve a final concentration of 10 mg/mL.

Antibacterial activity testing – determination of minimum inhibitory concentration (MIC) and minimum bactericidal concentration (MBC). Samples of each of these chemical compounds were reconstituted into an appropriate volume of DMSO to achieve a final concentration of 10 mg/mL. MIC values for these compounds was determined by two-fold broth microdilution in 96-well microtiter plates. Briefly, overnight cultures of *Escherichia coli* ATCC 25922, *Escherichia coli* 4, MRSA ATCC 43300 and MRSA 06/04 (see table S1 for further information about the isolates) were diluted in sterilised PBS to approximately 10^5 CFU/mL. Aliquots of 5 µL were then transferred to separate wells in a 96-well plate that contained 100 µL of each compound at varying concentrations (ranging from 100-0.195 µg/mL) prepared from two-fold serial dilutions in Mueller-Hinton (MH) broth. Plates were incubated at 37 °C for 18 hours using an Omnilog® automated incubator (Biolog Inc.;21124 Cabot Boulevard, Hayward, CA 94545,USA) and MIC values recorded.

Determination of the MBC values for all compounds tested above was performed in MH broth media. Again, 5µL were collected from the MICs 96-well plates (above) and re-inoculated into fresh sterile 96-well plates containing fresh MH. Plates were incubated under the same conditions mentioned above. The assay was performed in triplicate for each compound.

Table S1. UCD Centre for Food Safety strains used for determination of antibacterial activity.

	Strains	Type	Hospital/Isolation	Source	ARP (Resistant to)
Gram-negative	*E. coli* 25922	Reference	FDA strain Seattle 1946 [DSM 1103, NCIB 12210]	-	PEN; VAN; AMP; CLI; CL
	E. coli 4	Clinical isolate	UCD Veterinary Hospital	Bovine	AMC; AMP; C; CIP; F; Fc; Gm; N; NAL; S; Su; TET; TMP
Gram-positive	MRSA ATCC 43300	Reference	Kansas	Human	AMP; PEN; OXA; MET; AXO; CIP; LEVO; GAT; ERY; CLI
	MRSA 06/04	Clinical isolate	-	Human	AMP; PEN; OXA; MET; AXO; CIP; LEVO; GAT; ERY

Abbreviations: MRSA – Methicillin-resistant *Staphylococcus aureus*; ARP – Antibiotic resistant profile; AMC – Amoxicillin-Clavulanic acid; C – Chloramphenicol; F – Furazolidone; Fc – Florfenicol; Gm – Gentamycin; N – Neomycin; NAL – Nalidixic acid; S – Streptomycin; Su – Sulfonamides; TET – Tetracycline; TMP – Trimetoprim; AMP – Ampicillin; PEN – Penicillin; OXA – Oxacillin; MET – Methicillin; AXO – Ceftriaxone; CIP – Ciprofloxacin; LEVO – Levofloxacin; GAT – Gatifloxacin; ERY – Erythromycin; CLI – Clindamycin; CL – Cephalexin; VAN - Vancomycin.

Table S2. Antibacterial activity of compounds tested – MIC and MBC results (triplicates).

Compound	*E. coli* 25922		*E. coli* 4		MRSA ATCC 43300		MRSA 06/04	
	MIC[b]	MBC	MIC	MBC	MIC	MBC	MIC	MBC
**3**	>100	>100	>100	>100	>100	>100	>100*	>100
**4**	>100	>100	>100	>100	>100	>100	>100*	>100
**5**	>100	>100	>100	>100	**12.5**	**12.5**	**12.5**	**50**
**6**	>100	>100	>100	>100	>100	>100	>100*	>100
**S1**	>100	>100	>100	>100	**25**	**25**	**25**	**50**
**7**	>100	>100	>100	>100	**50**	**50**	**50**	**50**
**8**	>100	>100	>100	>100	**50**	**100**	**50**	**50**

* denotes a change in strain phenotype [b] MIC – minimum inhibitory concentration, MBC – minimum bactericidal concentration. Values are given in mg/L. Bold denotes compounds that showed activity against the tested strains. The maximum concentration of compounds tested was 100 mg/L.

The intermediates in the synthesis of **3**-**6** were also tested and it was observed one compound, **S1**, displayed anti-MRSA activity and was stable for the duration of the assay. However, **5** was found to be more potent.

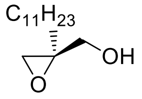

S1
